# Faba bean and pea harvest index estimations using aerial-based multimodal data and machine learning algorithms

**DOI:** 10.1093/plphys/kiad577

**Published:** 2023-11-03

**Authors:** Yishan Ji, Zehao Liu, Yuxing Cui, Rong Liu, Zhen Chen, Xuxiao Zong, Tao Yang

**Affiliations:** National Key Facility for Crop Gene Resources and Genetic Improvement/Institute of Crop Sciences, Chinese Academy of Agricultural Sciences, Beijing 100081, China; National Key Facility for Crop Gene Resources and Genetic Improvement/Institute of Crop Sciences, Chinese Academy of Agricultural Sciences, Beijing 100081, China; National Key Facility for Crop Gene Resources and Genetic Improvement/Institute of Crop Sciences, Chinese Academy of Agricultural Sciences, Beijing 100081, China; National Key Facility for Crop Gene Resources and Genetic Improvement/Institute of Crop Sciences, Chinese Academy of Agricultural Sciences, Beijing 100081, China; Institute of Farmland Irrigation, Chinese Academy of Agricultural Sciences, Xinxiang 453002, China; National Key Facility for Crop Gene Resources and Genetic Improvement/Institute of Crop Sciences, Chinese Academy of Agricultural Sciences, Beijing 100081, China; National Key Facility for Crop Gene Resources and Genetic Improvement/Institute of Crop Sciences, Chinese Academy of Agricultural Sciences, Beijing 100081, China

## Abstract

Early and high-throughput estimations of the crop harvest index (HI) are essential for crop breeding and field management in precision agriculture; however, traditional methods for measuring HI are time-consuming and labor-intensive. The development of unmanned aerial vehicles (UAVs) with onboard sensors offers an alternative strategy for crop HI research. In this study, we explored the potential of using low-cost, UAV-based multimodal data for HI estimation using red–green–blue (RGB), multispectral (MS), and thermal infrared (TIR) sensors at 4 growth stages to estimate faba bean (*Vicia faba* L.) and pea (*Pisum sativum* L.) HI values within the framework of ensemble learning. The average estimates of RGB (faba bean: coefficient of determination [*R*^2^] = 0.49, normalized root-mean-square error [NRMSE] = 15.78%; pea: *R*^2^ = 0.46, NRMSE = 20.08%) and MS (faba bean: *R*^2^ = 0.50, NRMSE = 15.16%; pea: *R*^2^ = 0.46, NRMSE = 19.43%) were superior to those of TIR (faba bean: *R*^2^ = 0.37, NRMSE = 16.47%; pea: *R*^2^ = 0.38, NRMSE = 19.71%), and the fusion of multisensor data exhibited a higher estimation accuracy than those obtained using each sensor individually. Ensemble Bayesian model averaging provided the most accurate estimations (faba bean: *R*^2^ = 0.64, NRMSE = 13.76%; pea: *R*^2^ = 0.74, NRMSE = 15.20%) for whole growth stage, and the estimation accuracy improved with advancing growth stage. These results indicate that the combination of low-cost, UAV-based multimodal data and machine learning algorithms can be used to estimate crop HI reliably, therefore highlighting a promising strategy and providing valuable insights for high spatial precision in agriculture, which can help breeders make early and efficient decisions.

## Teaser

Multiple affordable aerial-based sensors can be used to estimate the harvest index of faba bean and pea with an ensemble Bayesian model averaging algorithm.

## Introduction

Food legumes and cereals are the 2 main crop types that have been domesticated and consumed by humans. Food legume crops are high in protein, moderate in starch, and low in fat and can thus be used as a supplement to other major carbohydrate sources in human nutrition (Broughton et al. 2003). Faba bean (*Vicia faba* L.) and pea (*Pisum sativum* L.) are 2 representative crops of food legumes that are popular and widely cultivated worldwide. According to the FAO statistics in 2021 (FAOSTAT; http://www.fao.org/), faba bean is grown in over 70 countries and pea in over 90 countries worldwide. These are important sources of plant protein for humans and livestock, with high nutritional value, versatility, and profitability (Garousi et al. 2017). The rhizobia in their root systems can also fertilize the soil via biological nitrogen fixation, which contributes to sustainable land use and plantation industry development (Beringer et al. 1979).

The harvest index (HI) was created in the early 1960s to represent the ratio of grain yield to biological yield or above-ground biomass (AGB) (Donald 1962). As a critical biological parameter, the HI is of great importance in crop breeding, growth simulation, and cultivation management and has played an important role in the “Green Revolution” of the 1960s (Dong et al. 2023). Timely and accurate HI values for crops are therefore crucial for breeders and agricultural management departments, which can, in turn, assist with the screening of optimal varieties, evaluation of growth status, improvement of cultivation techniques, and so on. However, HI research remains relatively limited compared with other crop traits, such as yield and biomass. The traditional approach for measuring the HI is based on destructive field techniques, in which the HI is obtained based on the ratio of crop yield to biomass. Although this method is highly accurate, it is time-consuming and labor-intensive, thereby making its implementation difficult for large-scale field studies (Ren et al. 2022). As an alternative, some studies have attempted to estimate the HI through the functional relationship between HI and time or remote sensing data based on satellite platforms (Sadras and Connor 1991; Moriondo et al. 2007).

Remote sensing data obtained from satellites have been shown to be feasible in agricultural research; however, applications are generally hindered by their low resolution and fixed revisit time (Wang et al. 2018; Cheng et al. 2022). As an alternative, unmanned aerial vehicles (UAVs) have become increasingly used by agricultural researchers for field data collection owing to their low cost, simple operation, and flexibility and because the load sensors can provide high spatial and temporal resolution images. Some crop traits have been successfully estimated using UAV-based data, including the emergence rate, plant height (PH), leaf area index (LAI), AGB, and yield (Jin et al. 2017; Liu et al. 2021; Ji et al. 2023). However, crop HI estimations based on UAV data have rarely been addressed in the literature. In fact, at the time of writing, only one study reported crop HI estimations based on UAV data (Ren et al. 2022), and no studies have published relevant reports on the HI estimation of faba bean or pea. This previous study used a UAV hyperspectral sensor to achieve a highly accurate HI estimation of winter wheat, but the approach is costly. In contrast, low-cost sensors have been the focus of broader research, including red–green–blue (RGB), multispectral (MS), and thermal infrared (TIR) sensors. For instance, Ji et al. (2023) successfully used an RGB sensor to estimate the AGB and yield. Fei et al. (2021) used an MS sensor to predict wheat grain yield and achieved satisfactory results. The fusion of data obtained from different sensors can also complement the range of variable information obtained using a single sensor, thereby improving the crop trait estimation accuracy. The potential of using fusion data from multiple affordable sensors is therefore worth exploring for HI estimation.

Previous studies have estimated many crop traits using machine learning algorithms including partial least squares regression (PLSR; Liu et al. 2021), random forest (RF; Leroux et al. 2019), support vector machine (SVM; Ji et al. 2022), ridge regression (RR; Fei et al. 2023), elastic net (EN; Fei et al. 2021), and k-nearest neighbor (KNN; Feng, Zhang, et al. 2020). However, these algorithms have their own merits under different conditions. For example, PLSR has been shown to provide a better estimate of maize LAI than SVM and RF (Liu et al. 2021), while RF performed better estimates of soil moisture content than PLSR and KNN (Cheng et al. 2022). The EN has exhibited the best AGB estimate ability at early filling and mid-filling stages but a relatively poorer estimate than RR at the podding stage (Ji et al. 2023). Ensemble learning (EL) has been proposed to improve the model estimation accuracy. The core of EL is to combine multiple basic learners to improve the generalization of the model (Zhou 2012). Compared with traditional machine learning methods, EL provides higher estimation accuracy by secondary learning of the output results from basic models (Zhang et al. 2019). EL is considered to be the state-of-the-art solution to tackle many challenges in machine learning, where the predictive performance of a single model can be improved by training multiple models and combining their predictions (Sagi and Rokach 2018). Mercedes et al. (2017) demonstrated the utility of EL in predicting topsoil texture with different source data sets, spatial coverage, and methodological approaches. Gyamerah et al. (2020) proposed a probabilistic forecasting model that utilizes quantile RF and the Epanechnikov kernel function. They applied this model to estimate groundnut and millet yield in Ghana and achieved greater accuracy in their estimations compared with the 4 base models. In addition, the EL has been employed to monitor PM2.5 (Feng, Li, et al. 2020), map forest changes (Clinton et al. 2015), predict alfalfa yield (Feng, Zhang, et al. 2020), and more. As a kind of EL, the ensemble Bayesian model averaging (EBMA) has been widely used in many studies, including weather forecasting, groundwater storage estimation, presidential election prediction, and rice phenology simulations (Raftery et al. 2005; Montgomery et al. 2015; Gao et al. 2021; Yin et al. 2021). However, few studies have reported crop trait estimation using EBMA, and few studies have used UAV-based multimodal data to estimate HI.

Considering the above discussion, this study uses 3 types of sensors (RGB, MS, and TIR) and machine learning to estimate the HI of faba bean and pea. This study aimed to (i) evaluate the HI estimated performance based on single and multiple sensors, (ii) compare the estimation accuracy of RF, SVM, EN, RR, and EBMA algorithms, and (iii) explore the ability to estimate HI at different growth stages.

## Results

### HI estimation for different sensors

In this study, the RGB, MS, TIR, and different combinations of sensors were used to estimate the HI for faba bean and pea. The data from 4 growth stages were analyzed to explore the ability of the different sensors to estimate the HI. The details of the estimation results are shown in Supplemental Tables S1 and S2. Overall, the multimodal data fusion exhibited better estimated performance than single sensors for both faba bean and pea.

**Figure 1.**
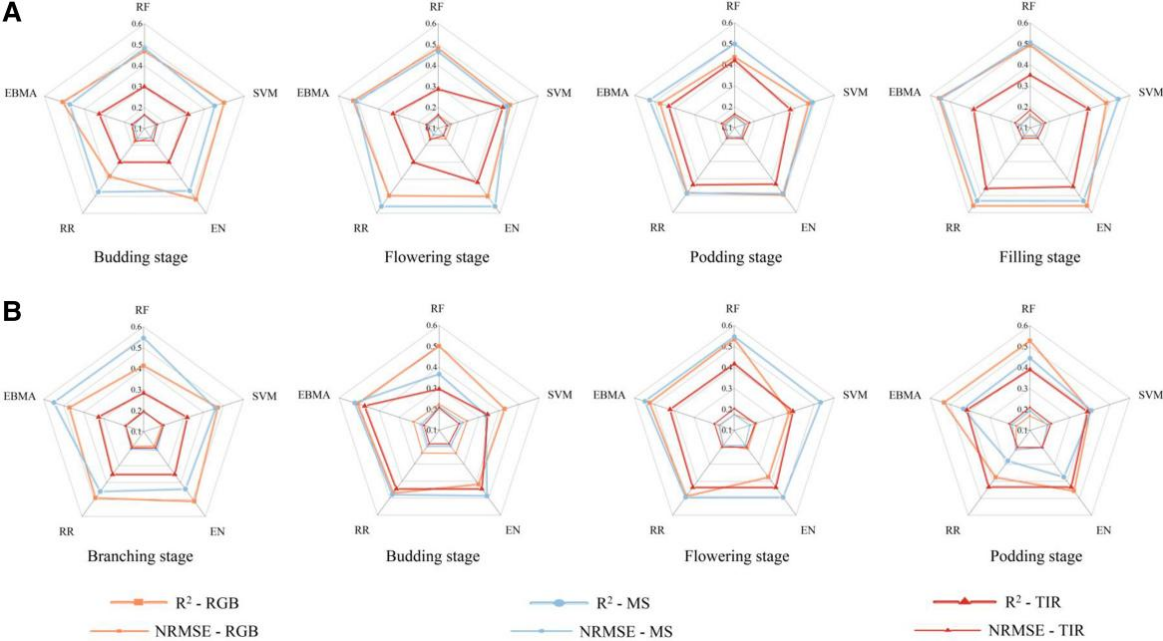
The estimated performance of single sensor. **A)** The estimated performance of single sensor in faba bean. **B)** The estimated performance of single sensor in pea.

In the case of using a single sensor (Fig. 1), the RGB, MS, and TIR sensors all contributed to the estimation of HI. Among them, the RGB and MS provided higher estimation accuracy, and both were comparable, while TIR provided relatively poorer estimation accuracy. For faba bean (Fig. 1A), the TIR (*R*^2^ = 0.29 to 0.46) provided the worst estimation, while RGB (*R*^2^ = 0.38 to 0.56) and MS (*R*^2^ = 0.44 to 0.56) performed better, showing little difference in their HI estimation accuracy for faba bean. The RGB obtained the best accuracy when using the EN and RR algorithms at the filling stage (*R*^2^ = 0.56), the MS obtained the highest *R*^2^ value (0.56) using RR at the flowering stage, and the best estimated accuracy of TIR was obtained at the filling stage using RR (*R*^2^ = 0.46). For pea (Fig. 1B), the TIR (*R*^2^ = 0.28 to 0.44) had the poorest estimated performance, while RGB (*R*^2^ = 0.37 to 0.53) and MS (*R*^2^ = 0.28 to 0.55) performed better, both exhibiting merits in the different growth stages when estimating the HI for pea. The RGB obtained the best estimation accuracy using RF at the flowering stage (*R*^2^ = 0.53), the MS achieved the highest *R*^2^ (0.55) using EBMA at the branching stage, and the best estimated accuracy of TIR was obtained at the flowering stage using EN (*R*^2^ = 0.44).

**Figure 2.**
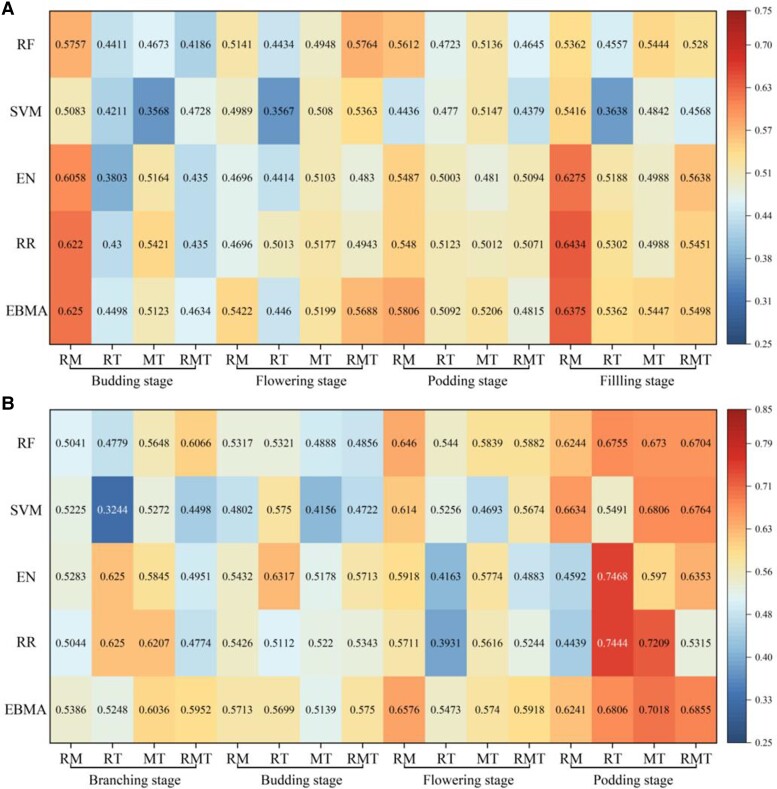
The estimated performance (*R*^2^) of multiple sensors. **A)** The estimated performance (*R*^2^) of multiple sensors in faba bean. **B)** The estimated performance (*R*^2^) of multiple sensors in pea. RM: RGB + MS; RT: RGB + TIR; MT: MS + TIR; RMT: RGB + MS + TIR.

In the case of using different combinations of sensors (Fig. 2), the RM (RGB + MS), RT (RGB + TIR), MT (MS + TIR), and RMT (RGB + MS + TIR) were employed for estimating the HI using the 5 selected machine learning algorithms. Compared with the single sensor, the multiple sensors improved the estimation accuracy of HI to a certain extent. For faba bean (Fig. 2A), RM achieved the best estimation accuracy (*R*^2^ = 0.64) when using the EBMA at the filling stage, RT provided the best estimation (*R*^2^ = 0.54) with the EBMA at the filling stage, MT obtained the highest *R*^2^ (0.54) using the EBMA at the filling stage, and the best estimated accuracy of the RMT was obtained at the flowering stage with RF (*R*^2^ = 0.58). The optimal estimation based on the fusion of multiple sensor data (*R*^2^ = 0.64) was improved by 0.08, 0.08, and 0.18 compared with the best estimations of RGB, MS, and TIR individually, respectively. For pea (Fig. 2B), RM achieved the best estimation accuracy (*R*^2^ = 0.66) when using SVM at the podding stage, RT provided the best estimation (*R*^2^ = 0.75) with EN at the podding stage, MT obtained the highest *R*^2^ (0.72) using RR at the podding stage, and the best estimated accuracy of RMT was obtained at the podding stage with EBMA (*R*^2^ = 0.69). The optimal estimation based on the fusion of multiple sensor data (*R*^2^ = 0.75) was improved by 0.22, 0.2, and 0.31 compared with the best estimations of RGB, MS, and TIR individually, respectively.

### HI estimation using different models

**Figure 3.**
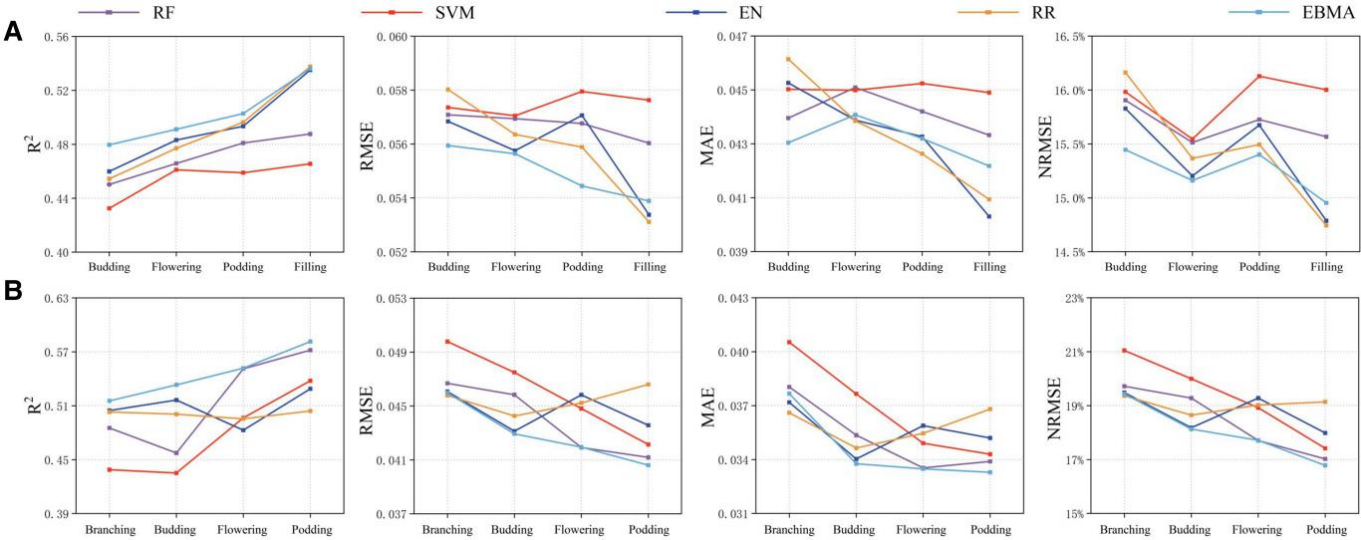
HI estimated performance of different models. **A)** HI estimated performance of different models in faba bean. **B)** HI estimated performance of different models in pea.

In this study, 4 base models (RF, SVM, EN, and RR) and an ensemble model (EBMA) were adopted to estimate the HI. Compared with the individual models, the EL model was able to improve the generalization by integrating multiple models, thus providing a more stable estimation result. Figure 3 presents the mean values of *R*^2^, RMSE, mean absolute error (MAE), and normalized root-mean-square error (NRMSE) of the HI for faba bean and pea, which were obtained based on 7 types of data (single sensor and multiple sensors). Overall, the EBMA performed better for estimating the HI than the base models for both faba bean and pea, providing higher *R*^2^ values and lower RMSE, MAE, and NRMSE values.

For the estimation of faba bean HI (Fig. 3A), the *R*^2^ values of the base models were lower than the EBMA model except for the RR algorithm at the filling stage. Among the 4 base models, EN provided the best estimated performance at the budding and flowering stages, with *R*^2^ values of 0.46 and 0.48, respectively, and RR provided the best estimation at the podding and filling stages with *R*^2^ values of 0.46 and 0.48, respectively. The SVM was the poorest algorithm for estimating the faba bean HI at the 4 growth stages (*R*^2^ = 0.43, 0.46, 0.46, and 0.47). Compared with the best-performing base models, the EBMA model improved the *R*^2^ value from 0.46 to 0.48 at the budding stage, 0.48 to 0.49 at the flowering stage, and 0.49 to 0.5 at the podding stage. However, the EBMA failed to provide the best estimated accuracy at the filling stage, which was provided by RR. For the estimation of pea HI (Fig. 3B), the *R*^2^ values of the 4 base models were all lower than the EBMA model. Among the 4 base models, the EN algorithm provided the best estimation performance at the branching and budding stages, with *R*^2^ values of 0.5 and 0.52, respectively, while the RF algorithm performed the best at the flowering and podding stages, with *R*^2^ values of 0.55 and 0.57, respectively. The SVM produced the poorest estimation performance at the branching and budding stages, both with *R*^2^ values of 0.44, EN produced the poorest estimation performance at the flowering stage (*R*^2^ = 0.48), and RR produced the poorest estimation at the podding stage (*R*^2^ = 0.5). Compared with the best-performing base models, the EBMA model improved the *R*^2^ value from 0.5 to 0.52 at the branching stage, 0.51 to 0.53 at the budding stage, 0.55 to 0.56 at the flowering stage, and 0.57 to 0.58 at the podding stage.

These results demonstrate that the EBMA model performed better than the base models in estimating the HI of faba bean and pea. Although the EBMA model failed to provide the best estimation for all tests, it achieved a more stable performance by minimizing the deviations and randomness of the base models.

### Posterior weights of each base model

**Figure 4.**
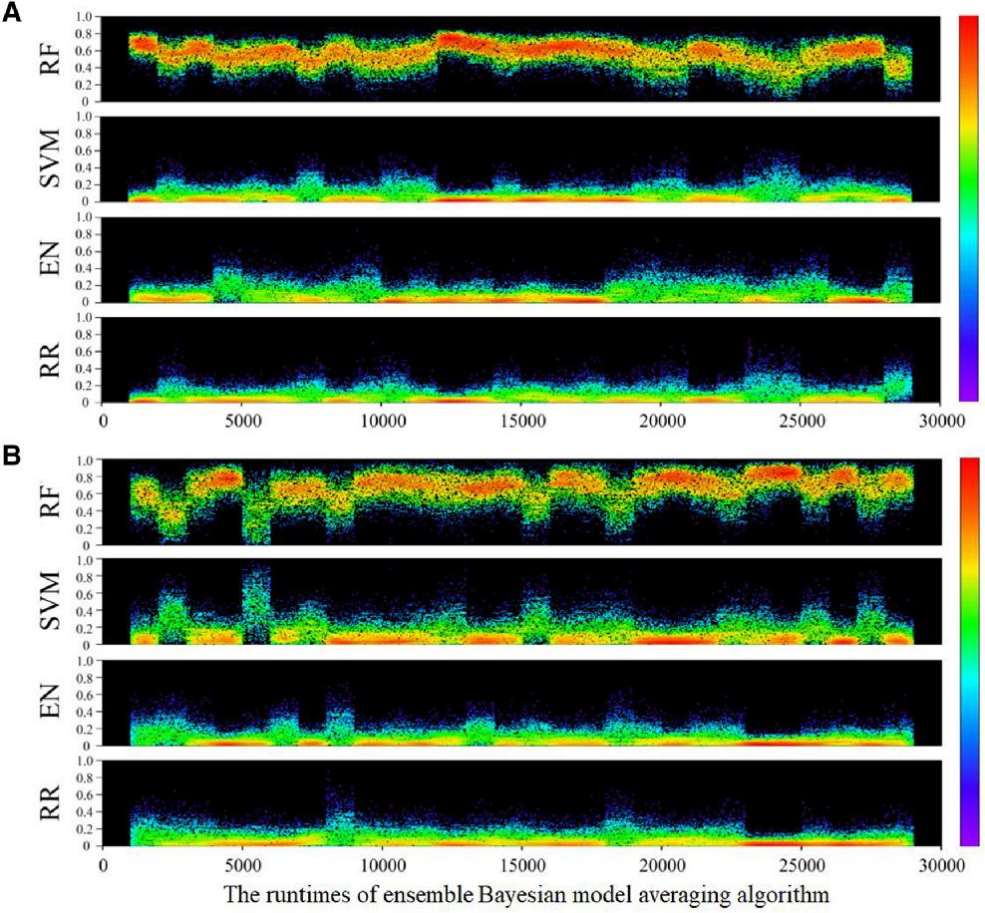
The posterior weights of each base model. **A)** The posterior weights of each base model in faba bean. **B)** The posterior weights of each base model in pea.

In this study, the EBMA model provided a more stable estimation performance by combining the advantages of each base model and avoiding overfitting or overestimating. In each HI estimation for faba bean and pea, 4 base models obtained 1,000 posterior weights, which were assigned by the ensemble model. A total of 28,000 posterior weights were generated for each base model, the distributions of which are presented as density dot graphs in Fig. 4 for faba bean and pea, respectively. In Fig. 4, the *X* axis represents the number of runs of the EBMA model, and the *Y* axis represents the size of the posterior weights assigned to each base learner. A larger weight indicates that the base learner has higher authority and confidence in generating the final prediction, which suggests that the base learner is considered more reliable and trustworthy. This can be attributed to its strong performance on the training data or its higher accuracy in a particular situation. In summary, larger weights assigned to a specific base learner in the EBMA model indicate that the learner is deemed more important and contributes substantially to the overall model.

In terms of the posterior weights of each base model, RF was assigned the highest weight, with a mean above 0.58, for both the faba bean and pea HI estimates. As illustrated in Fig. 4A, when estimating the HI of faba bean, the RF algorithm was assigned the highest posterior weights with a mean value of 0.5862, followed by the EN and RR algorithms with mean values of 0.1491 and 0.1476, respectively. In contrast, the posterior weights of the SVM algorithm were the lowest, with a mean value of 0.1171. As shown in Fig. 4B, when estimating the pea HI, the highest posterior weights were assigned to RF with a mean value of 0.5862, followed by SVM with a mean value of 0.1635, and the EN and RR algorithms obtained the lowest posterior weights with mean values of 0.1016 and 0.0973, respectively.

### HI estimation in different growth stages

The UAV-based multimodal data for whole growth stages were treated as a new data set to assess the effectiveness of the fusion of all growth stages for estimating HI. Compared with single growth stage, the whole growth stage provided the best estimates (faba bean: *R*^2^ = 0.57, NRMSE = 14.04%; pea: *R*^2^ = 0.54, NRMSE = 17.77%).

**Figure 5.**
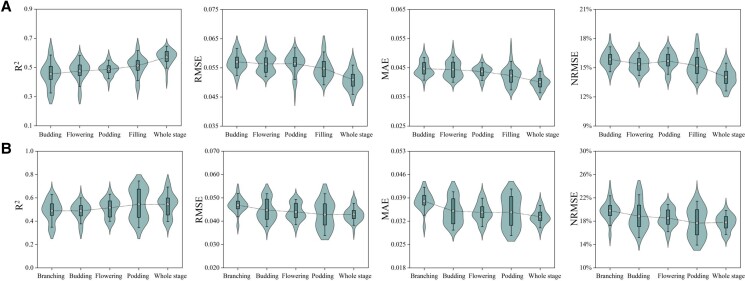
The estimated performance at different growth stages. **A)** The estimated performance of faba bean at different growth stages. **B)** The estimated performance of pea at different growth stages.

Figure 5A shows that the HI estimation accuracy for faba bean gradually increased with the advancing growth period. For example, the average *R*^2^ increased from 0.46 to 0.51, the average RMSE decreased from 0.057 to 0.0548, the average MAE decreased from 0.0447 to 0.0423, and the average NRMSE decreased from 15.86% to 15.21%. When the remote sensing data from four growth stages were fused into a new data set to estimate the HI for faba bean, the most accurate value was obtained with an *R*^2^ value of 0.57, which was 0.06 higher than that of the best single growth stage (filling stage). Figure 5B indicates that the accuracy of the pea HI estimates also gradually increased with the progressive growth stage, with average *R*^2^ values increasing from 0.49 to 0.54, average RMSE decreasing from 0.0469 to 0.0428, average MAE decreasing from 0.038 to 0.0334, and the average NRMSE decreasing from 19.81% to 17.75%. When fusing the remote sensing data of the 4 growth stages into a new data set to estimate the pea HI, the obtained estimation accuracy was slightly higher than that of the best single growth stage, which failed to improve the accuracy of the HI estimates as substantially as it did for faba bean.

### HI estimation map for optimal result

**Figure 6.**
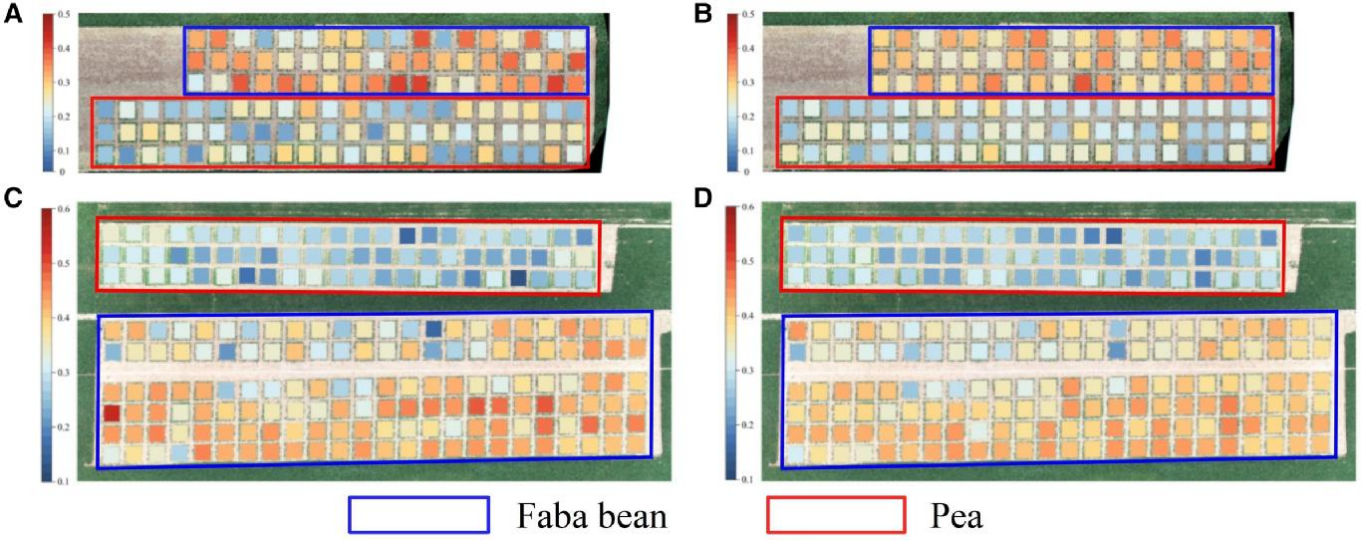
The distribution of HI. **A)** The measured HI in 2021. **B)** The estimated HI using EBMA in 2021. **C)** The measured HI in 2022. **D)** The estimated HI using EBMA in 2022.

In this section, the measured and best estimated HI values obtained from the EBMA model are used to draw Fig. 6, which intuitively presents the spatial distribution of faba bean and pea HI values in the field. The validation results corresponding to Fig. 6 are shown in the form of a scatter plot in Supplemental Fig. S1. Figure 6, A and C shows the distribution of measured HI values in 2021 and 2022, respectively, and Fig. 6, B and D shows the distribution of the best estimated HI values in 2021 and 2022, respectively.

The HI values obtained from the EMBA model are in good agreement with the measured HI values for faba bean and pea, which reflects a satisfactory estimation result. Furthermore, both the measured and estimated faba bean HI values were higher than those of pea in 2021 and 2022, which may be related to differences in their growth morphologies.

## Discussion

### Differences in HI estimation metrics for different crops

Faba bean and pea are crucial cool-season food legume crops that play important roles in food supply, agricultural economics, soil improvement, and ecosystem function. These crops offer both nutritional and economic benefits to humans, while also contributing to sustainable agricultural practices and ecosystem health. However, compared with popular grain crops (rice, wheat, and maize), there have been relatively fewer high-throughput plant phenotyping studies conducted on faba bean and pea. Therefore, the current study, which focuses on estimating the HI of faba bean and pea through high-throughput plant phenotyping studies in the field, is of great importance for the future development of cool-season legume crops. High-throughput plant phenotyping allows for the rapid and accurate measurement of various plant traits, which can aid in understanding the plant's growth, development, and response to environmental conditions. By estimating the HI of faba bean and pea, this study can provide valuable insights into their yield potential and resource allocation patterns, aiding in the improvement of cultivation practices and crop breeding strategies.

Figures 1 and 2 (single sensor and multiple sensors, respectively) present the HI estimation results in faba bean and pea, respectively. Overall, the HI estimation results in this study were acceptable for all growth stages of faba bean and pea. It is worth noting that the average estimation accuracy of pea was higher than that of faba bean at the same growth stage (budding, flowering, and podding). This can be caused by the following reasons. First, faba bean has higher levels of canopy coverage (CC) than pea at the same growth stage, and high levels of CC may lead to saturation of the optical sensors (Hoffmann et al. 2016; Rischbeck et al. 2016), which increases the estimation error of the model and produces estimates with relatively low accuracy. Second, different machine learning algorithms are applicable to the estimation of different crops with different phenotypic parameters (Fei et al. 2023), and the algorithm chosen in this study may be more applicable to pea, leading to better estimation accuracy. Third, the fact that pea and faba bean have different species characteristics may also lead to differences in estimation accuracy. Pea may have more obvious growth characteristics, such as leaf shape and size and branching, which help to accurately estimate HI by appearance and growth. Faba bean plants, on the other hand, are relatively large and may be relatively more subtle or less variable in these characteristics, making the estimation process more difficult.

### Comparison of HI estimates based on different sensors

Numerous previous studies have presented the feasibility of low-cost sensors for estimating crop phenotypic traits, and that the fusion of data from multiple sensors can provide better estimation results (Feng, Zhou, et al. 2020; Zhang et al. 2023). However, HI estimations based on low-cost sensors have not been previously investigated.

As single sensors, the RGB and MS showed little difference in the HI estimation. Previous studies generally preferred MS for estimating crop yield and ignored RGB, possibly because the vegetation indices (Vis) derived from the MS sensor are more sensitive to yield. Maimaitijiang et al. (2020) reported that RGB-derived canopy structure information could potentially replace VIs to some extent. The sensitivity between the HI and RGB-derived canopy structure information (PH and CC) is similar to the MS-derived Vis. Thus, they tended to be consistent in the HI estimation of faba bean and pea in the current study. Compared with RGB and MS, the TIR sensor provided the worst estimates. Although TIR had a lower contribution to the HI estimation, it can potentially serve as a component of multisensor data for estimating faba bean and pea HI.

When using multiple sensors, the HI estimates were more accurate and stable than those obtained using a single sensor. This result is in great agreement with previous studies that demonstrated that combining data from multiple sensors could improve crop trait estimation, such as sorghum biomass and cotton yield (Feng, Zhou, et al. 2020; Li et al. 2022). This is likely related to the fact that the variables provided by the 3 sensors are independent of each other and thereby provide complementary and unique information, which is beneficial for improving HI estimations (Rischbeck et al. 2016). In addition, the inclusion of texture information (TI) from three sensors could provide spatial canopy architecture information (Colombo et al. 2003), which may also improve the estimation accuracy, as has been reported for soybean yield estimation (Maimaitijiang et al. 2020). However, as shown in Fig. 2, the fusion effects of using all 3 sensors were not necessarily higher than those obtained using 2 sensors, which may be related to data redundancy between the 3 sensors. If these sensors provide similar or duplicate information, fusing these data may not substantially improve accuracy. Conversely, redundancy may increase noise and uncertainty, thereby reducing the accuracy of the fused data. As previously shown by Maimaitijiang et al. (2017), the canopy temperature information and spectral information can be prone to information overlap, thereby affecting the estimation ability.

### Performance of machine learning algorithms in HI estimation

Crop phenomic research is rapidly developing, and the application of machine learning in agricultural research has become a hot topic (Rehman et al. 2018). The EL method takes advantage of the complementary strengths of basic models to minimize random errors and thus provide more reliable estimates. As a powerful framework, the EBMA can combine the basic models in a principled way to improve the estimation accuracy, and it has been used to estimate a wide range of processes, including presidential elections, the occurrence of insurgencies, and wheat yield (Montgomery et al. 2011, 2015; Fei et al. 2023).

The EBMA approach was selected for estimating crop HI due to its ability to effectively handle uncertainties that may arise during the estimation process. EBMA, as a powerful framework in EL, can produce better estimates from its ensemble members. In accordance with previous studies, most EBMA results obtained a higher estimation accuracy than the basic models (Shu et al. 2022). However, there were still some basic models that outperformed EBMA in terms of estimation accuracy in this study. The reasons behind this could be attributed to the following factors. Firstly, the EBMA operated by combining the estimates of multiple basic models to derive the final estimation result. However, if there were substantial differences or conflicts between the basic models, this could have adversely affected the performance of EBMA. Secondly, the performance of EBMA also highly relied on the diversity exhibited by the basic models. If the basic models lacked diversity and consistently produced similar errors or biases during the prediction process, it could have imposed limitations on the effectiveness of the EBMA (Feng, Li, et al. 2020; Feng, Zhang, et al. 2020; Fei et al. 2023). Although the EBMA did not always perform best, it minimized the bias and randomness of the basic models, making it more stable. In addition, the EBMA also differs from stacking EL in that the former produces weighting coefficients that are all positive, whereas the latter produces regression coefficients that are both positive and negative, and the negative regression coefficients can be difficult to interpret in some cases (Raftery et al. 2005). The EBMA, therefore, offers an alternative strategy for crop trait estimation using EL.

### HI estimation in different growth stages

This study used UAV-based multimodal data from 4 key growth stages to estimate the HI. Overall, the HI estimation accuracy progressively increased with growth stage. Although the relationship between HI estimation accuracy and growth stage has not been directly reported, it can be explored from previous studies on biomass and yield. For instance, Che et al. (2022) demonstrated that late growth stages achieved better AGB estimation accuracy, and Hassan et al. (2019) revealed that the grain-filling stage could provide better estimation accuracy for wheat yield than other growth stages. In the current study, the last growth stage (i.e. filling stage for faba bean; podding stage for pea) was found to provide the best estimation accuracy compared with those of the other three growth stages, which is consistent with previous results. It is worth noting that the improvement of accuracy in the filling stage is considerably higher than that of other growth stages (Fig. 5A). This may be because the canopy structure of crops tends to stabilize during the filling stage, which makes certain variables (e.g. PH, VIs, CC, TI, and normalized relative canopy temperature [NRCT]) extracted based on remote sensing images more suitable for estimation. Additionally, there are several reasons why whole growth stages are more accurate than single growth stage in estimating HI. Firstly, by including data from multiple growth stages, the model can take into account the physiological changes and environmental adaptations that occur in plants at different stages. This holistic approach enhances the accuracy of the estimation model for HI. Secondly, with the broader range of data and more information available in the whole growth stages, the model can better capture the variations and complexities of plant growth, thus providing more accurate estimates. Finally, different growth stages may interact and influence each other during plant growth. By utilizing a complete growth stages data set, it becomes possible to capture the combined effects of these interactions, resulting in more accurate estimations. The time series data should be collected in the future to make a dynamic estimate of HI for assessing dynamic crop growth information and determining the optimal time for early HI estimations.

### Future improvements

Although the results are encouraging, there are still some improvements that should be made in future work. The HI estimation accuracy of the data fusion of 2 and 3 sensors in this study was higher than that of a single sensor owing to the complementary nature of the canopy structure features provided by RGB, the spectral features provided by MS, and the temperature information provided by TIR to some extent (Stanton et al. 2017). In addition, the estimation accuracy based on 3-sensor data did not always perform better than 2-sensor data. Therefore, improved sensor fusion methods and data screening strategies should be explored. The sample size used in this study also has some limitations. Future studies should include more genotypes and increase the crop field scale to test the applicability and robustness of the models.

Finally, recent studies have shown that deep learning can better mine data than traditional machine learning algorithms and improve the research accuracy (Wang et al. 2019; Niu et al. 2021). The inclusion of deep learning should also be considered to further explore the application ability of UAV-based multimodal data in agricultural research.

## Conclusions

This study explored the potential of using multiple low-cost, UAV-based sensors for the HI estimation of faba bean and pea within the framework of EL and investigated the effect of different growth stages on the HI estimation accuracy. The conclusions are summarized as follows.

1. In the case of faba bean and pea HI estimations based on a single sensor, the difference in estimation accuracy between MS and RGB was not substantial. Although TIR showed relatively poor estimation accuracy, it also exhibited potential for estimating the HI. In addition, although multisensor data fusion improved the estimation accuracy of HI to some extent, the estimation accuracy based on 3-sensor data was not always better than 2-sensor data.
2. Although 4 basic models (RF, SVM, EN, and RR) demonstrated some capability to estimate HI in this study, the accuracies and stabilities were poor. Compared with basic models, the EBMA contributed to higher estimation accuracy in most cases.
3. The HI estimation accuracy gradually improved with the advancing growth stage of faba bean and pea, and the fusion of data from the 4 growth stages provided a better estimated performance than that obtained using a single growth stage.

The results reported herein reflect the effectiveness of machine learning and multimodal UAV data for estimating faba bean and pea HI. This study provides valuable insights for accelerating the breeding process and different ideas for crop HI estimation. However, additional studies should be conducted on larger numbers of genotypes and at multiple experimental sites to test and verify the robustness and adaptability of the proposed method.

## Materials and methods

### Experimental site and design

**Figure 7.**
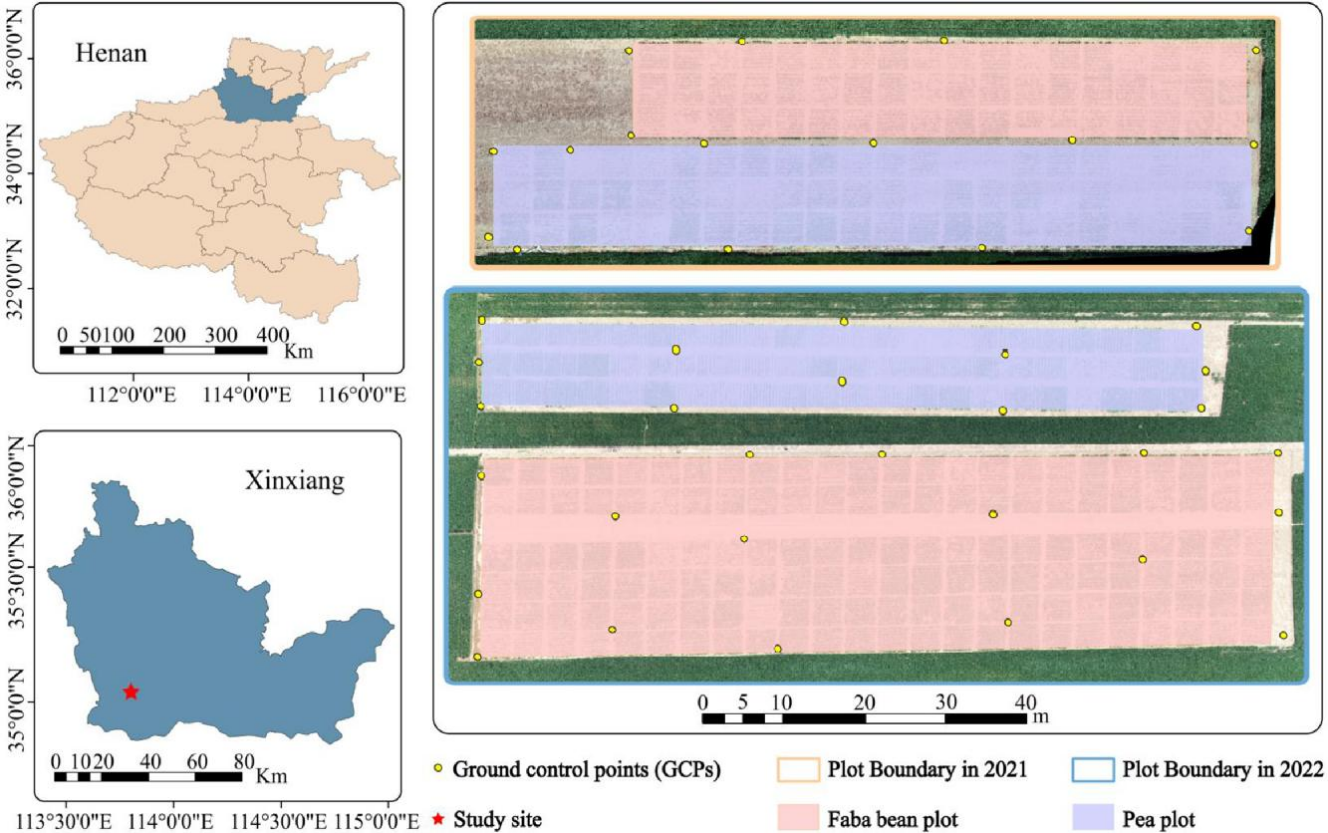
The geographical location of the research area.

The experiment was carried out over 2 years (2020 to 2021 and 2021 to 2022) at the experimental base of the Chinese Academy of Agricultural Sciences in Xinxiang City, Henan Province, China (Fig. 7). The coordinates of the site are longitude 113°45′38″E and latitude 35°8′10″N. During the first growing season (November 2020 to June 2021), there was a total rainfall of 171.81 mm and an average monthly temperature and humidity of 12.6 °C and 60.1%, respectively. During the second growing season (November 2021 to June 2022), there was a total rainfall of 68.69 mm, while the average monthly temperature and humidity were 12.1 °C and 60.9%, respectively.

The experimental setup was a randomized complete block design with 3 replications. Each plot had an area of 10 m^2^ (3.33 m long × 3 m wide). Spot sowing of faba bean (*V. faba* L.) and pea (*P. sativum* L.) was adopted in the 2-year experiment and was performed on November 15, 2020, and on November 17, 2021. Ten rows were planted in each plot, with 25 faba bean seeds or 50 pea seeds per row. In the first year, the experimental design consisted of 18 faba bean varieties and 22 pea varieties, which made up a total of 54 faba bean plots and 66 pea plots. In the second year, 30 faba bean varieties were added based on the first year, which also included 3 replications. The experiment in the second year, therefore, contained 144 faba bean plots and 66 pea plots. The management practices, including the application of pesticides, irrigation, and fertilizers, followed the local procedures.

### Data collection

#### Collection of field data

The HI is the ratio of yield and AGB (Equation 1). Three representative plants (faba bean or pea) in each plot were, therefore, collected at the maturity stage and were used to measure the yield and AGB. After cutting the roots of the faba bean or pea, all components were dried at 80°C for 96 h until achieving a constant weight. All components were weighed, and the AGB was obtained. All grains were weighed to obtain the yield. Table 1 presents the statistics of the field HI.


$$\mathrm{HI}=\;\frac{W_{Y}}{W_{\mathrm{AGB}}},$$ (1)

where HI is the harvest index, $W_{Y}$ is the measured yield, and $W_{\mathrm{AGB}}$ is the measured above-ground biomass.

**Table 1.** Descriptive statistics for field-measured HI

Year	Crops	No. of samples	Mean	Min.	Max.	SD	CV (%)
2020 to 2021	Faba bean	54	0.2984	0.1463	0.4079	0.0686	22.99
2020 to 2021	Pea	66	0.2099	0.1	0.3053	0.0605	28.83
2021 to 2022	Faba bean	144	0.3866	0.1509	0.5496	0.063	16.29
2021 to 2022	Pea	66	0.2657	0.1279	0.3438	0.0476	17.90

#### Collection of UAV data

**Figure 8.**
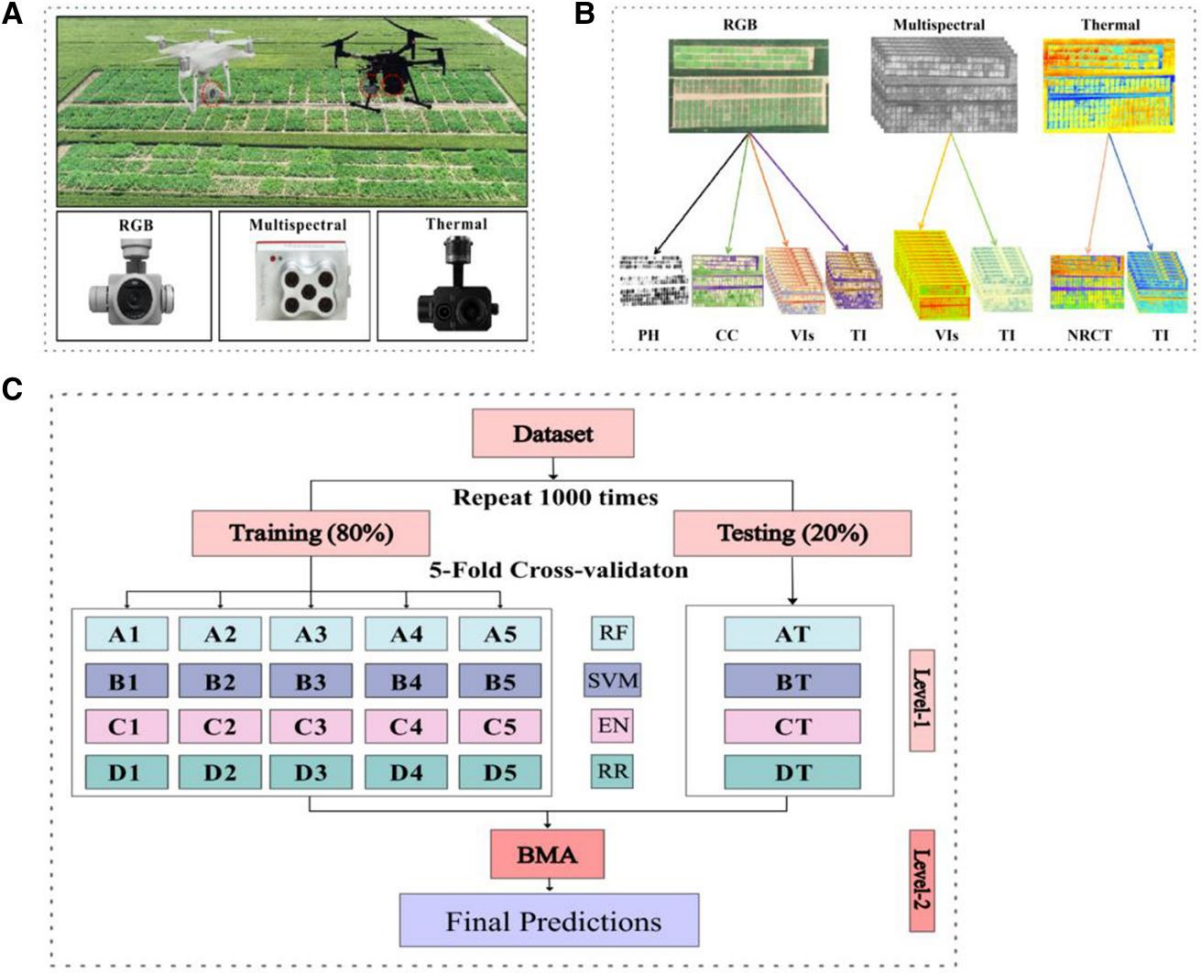
The presentation of observation platforms, sensors, features, and models. **A)** The observation platforms and 3 types of sensors. **B)** The different features extracted from 3 types of sensors. **C)** The workflow of model building for estimating HI.

In this study, 2 electric quadcopter UAVs were adopted as the observation platform. These included a DJI Phantom 4 Pro (SZ DJI Technology Co., Shenzhen, China) equipped with a Phantom camera and a DJI Matrice 210 (SZ DJI Technology Co., Shenzhen, China) equipped with a Red-Edge MX camera (MicaSense Inc., Seattle, USA) and a Zenmuse XT2 camera (SZ DJI Technology Co., Shenzhen, China). The observation platform and 3 sensors used in this study are shown in Fig. 8A.

The Phantom camera was used for collecting RGB images in jpg format. The Red-Edge MX camera was used for collecting MS images in tif format and could simultaneously collect images with 5 bands (blue, green, red, red edge, and near infrared). The Zenmuse XT2 camera was used for collecting TIR images in tiff format and recording the temperature in the 7.5 to 13.5 *μ*m spectral region with a 5 °C thermal sensitivity.

A total of 8 UAV flight campaigns were carried out in this study, and the detailed parameters are listed in Table 2. All campaigns were conducted under cloudless and low wind speed conditions between 11:00 and 13:00 to avoid errors caused by cloud cover and wind. The flight was conducted at an altitude of 30 m and a speed of 1.2 m/s. For the RGB camera, DJI GS Pro software was used to set both forward overlap and side overlap to 85%. For the MS and TIR cameras, both forward overlap and side overlap were set to 90% using DJI Pilot software. During the flight missions, the calibrated reflectance panel collected images both before and after each flight, which were later used to calibrate the MS images. Similarly, portable thermometers were used to measure the surface temperature of blackboards for TIR image calibration. A total of 16 and 28 ground control points (GCPs) were laid out in the first and second years of the experiment, respectively. They were set and recorded using a differential global navigation satellite system and remained fixed throughout the growing season.

**Table 2.** Parameters of each UAV flight

Crops	Date	Stage	Flight time (min)		Ground sample distance (cm)		
			RGB	MS/TIR	RGB	MS	TIR
Faba bean	2021/04/07	Budding	17.0	19.6	0.85	2.05	2.40
	2021/04/17	Flowering	17.3	20.0	0.82	2.00	2.38
	2021/04/28	Podding	17.2	19.4	0.74	2.05	2.42
	2021/05/08	Filling	16.8	19.6	0.84	2.11	2.77
	2022/04/11	Budding	16.8	19.6	0.81	2.00	2.49
	2022/04/18	Flowering	17.2	19.8	0.83	2.14	2.70
	2022/04/29	Podding	17.2	19.6	0.90	1.98	2.45
	2022/05/10	Filling	17.3	19.8	0.89	2.02	2.47
Pea	2021/04/07	Branching	17.0	19.6	0.85	2.05	2.40
	2021/04/17	Budding	17.3	20.0	0.82	2.00	2.38
	2021/04/28	Flowering	17.2	19.4	0.74	2.05	2.42
	2021/05/08	Podding	16.8	19.6	0.84	2.11	2.77
	2022/04/11	Branching	16.8	19.6	0.81	2.00	2.49
	2022/04/18	Budding	17.2	19.8	0.83	2.14	2.70
	2022/04/29	Flowering	17.2	19.6	0.90	1.98	2.45
	2022/05/10	Podding	17.3	19.8	0.89	2.02	2.47

### UAV image stitching

In this study, Pix4Dmapper 4.4.12 (Pix4D SA, Lausanne, Switzerland) was used to mosaic and orthorectify the RGB, MS, and TIR images. Several steps were conducted for the stitching process, including geolocating the images, importing the GCPs, aligning the images, building dense point clouds, generating a digital surface model (DSM), generating a digital terrain model (DTM), and obtaining orthomosaic and calibrating radiometric information.

For the MS image calibration, the Pix4Dmapper 4.4.12 automatically identified the calibrated reflectance panel images, read the reflectance values to create a band-response function of each band, and then used the band-response function to convert the digital number (DN) values of the MS images into reflectance values. For the TIR image calibration, the DN values of blackboards were extracted using ArcMap 10.5 (Environmental Systems Research Institute, Inc., Redlands, USA). A linear relationship between the DN values and actual temperature was derived using a portable thermometer, with the formula *T* = 0.2813 ∗ DN − 295.98. This relationship was then applied to convert the DN values of the TIR images into temperature values.

### Multimodal data extraction

Before extraction, several VIs, including the excess red index and excess green index, were used to construct a binary mask map for excluding the pixels of weeds and background soil in ArcMap 10.5. After using the mask, each image only contained pixels of faba bean or pea. Figure 8B shows the potential input variables from the three types of sensors.

### RGB data extraction

In this study, the RGB images were adopted to extract the PH, CC, TI, and DN values of each band. The PH was extracted from the crop surface model (CSM, Equation 2) and used as a feature for estimating HI.


CSM=DSM−DTM. (2)

The CC refers to the crop canopy vertical projection area as a proportion of the ground surface area and was calculated according to Equation 3 (Cheng et al. 2022).


$$\mathrm{Canopy}\;\mathrm{coverage}\;=P_{\mathrm{canopy}}/\;P_{\mathrm{total}},$$ (3)

where $P_{\mathrm{canopy}}$ is the number of faba bean or pea canopy pixels and $P_{\mathrm{total}}$ is the total number of pixels in the sampled plot.

The TI of each band was extracted using the gray level co-occurrence matrix (GLCM) in ENVI 5.3, which has been shown to be effective for estimating AGB and yield (Ji et al. 2023). Following previous work (Shu et al. 2022), the sliding window and sliding step were set as 7 × 7 and 2, respectively. The output of the GLCM calculation includes contrast, correlation, dissimilarity, entropy, homogeneity, mean, second moment, and variance in each plot. For a detailed description of the 8 texture metrics used, please refer to the study by Nichol and Sarker (2011).

The DN values were extracted using the same region of interests as the PH extraction. The DN values derived from each band were adopted to construct 10 VIs that have commonly been used for estimating the AGB or yield in previous studies. Supplemental Table S3 lists the specific VI formula and its sources.

### MS data extraction

The radiometrically calibrated MS images were used to extract the TI and reflectance values of each band. The TI and reflectance values from the MS band were extracted using the same methods as those for the RGB band in section "RGB data extraction". The reflectance values derived from the MS band were used to calculate 15 commonly used VIs. Supplemental Table S4 lists the specific VI formula and its sources.

### TIR data extraction

The calibrated TIR images were used to extract the TI and NRCT for estimating the HI. The TI values from the TIR band were extracted using the same methods as those for the RGB band in section 2.4.1. The NRCT has been successfully applied to estimate yield in a previous study (Maimaitijiang et al. 2020) but has not been explored to estimate HI. The NRCT was calculated according to Equation 4.


$$\mathrm{NRCT}\;=\;(T_{i}-T_{\min})\;/\;(T_{\max}-T_{\min}),$$ (4)

where $T_{i}$ is the temperature of pixel *i* and $T_{\min}$ ($T_{\max}$) is the minimum (maximum) temperature in the whole field.

### Modeling methods

This study obtained estimations using the RF (Breiman 2001), SVM (Cortes and Vapnik 1995), EN (Zou and Hastie 2015), RR (Tikhonov 1943) and EBMA models to explore the relationship between the UAV-based images and the HI of faba bean and pea. Although these algorithms have been successfully applied for crop trait estimations, the individual models are less stable owing to the influence of the model structure and parameters (Yin et al. 2021).

Bayesian model averaging (BMA) is a widely recognized method of calculating the weights of multiple models, which is a hypothesis based on Bayesian theorem. This approach takes into account the uncertainty associated with different models and uses it as a benchmark for calculating the weight of each model. By synthesizing these weights, BMA achieved more accurate estimation results. BMA is a method of synthesizing model results using statistical probability theory. In this method, *y* is assumed to be the predictor variable of interest, *D* = {*y*_1_, *y*_2_, …, *y_T_*} represents the measured data set, and *M* = {*M*_1_, *M*_2_, …, *M_K_*} represents the model space containing all possible prediction models. As a result, the expression for the posterior probability density function of *y* is as follows:


$$p\;(y/D)\;=\;\overset{k}{\underset{i\;=\;1}{\sum }}\;P\;(M_{i}/\;D)\;p\;(y\;/\;M_{i},\;D),$$ (5)

where $P\;(M_{i}\;/\;D)$ is the posterior probability of model $M_{i}$ and $p\;(y\;/\;M_{i},D)$ is the posterior distribution of the forecast *y* with known data *D* and model $M_{i}$.

From Equation 5, it can be seen that the posterior distribution of *y* is the mean value obtained by taking the posterior probability $P\;(M_{i}/\;D)$ of model $M_{i}$ as the weight and weighting the posterior distribution of model $p\;(y\;/\;M_{i},D)$. The effect belongs to variable weight estimation; i.e. the weights will change with the change of the model forecast accuracy, and the higher the forecast accuracy, the greater the weight will be given to the model, and vice versa, so as to improve the forecast accuracy of the combined forecast model.

The predicted mean and variance of the posterior distribution of *y* are calculated as follows:


$$E\;(y/D)\;=\;\overset{k}{\underset{i\;=\;1}{\sum }}\;P\;(M_{i}/\;D)\;E\;[p\;(y\;/M_{i},\;D)]\;=\;\overset{k}{\underset{i\;=\;1}{\sum }}\;\omega_{i}\;\eta_{i},$$ (6)

$$Var\;(y/D)\;=\;\overset{k}{\underset{i\;=\;1}{\sum }}\;\omega_{i}\;{\left(\eta_{i}\;-\;\overset{k}{\underset{i\;=\;1}{\sum }}\;\omega_{i}\;\eta_{i}\right)}^{2}\;+\;\overset{k}{\underset{i\;=\;1}{\sum }}\;\omega_{i}\;\sigma_{i}^{2}\;,$$ (7)

where $\omega_{i}=P\;(M_{i}\;/\;D)$, $\sum_{i\;=\;1}^{k}\omega_{k}=1$. $\eta_{i}$ and $\sigma_{i}^{2}$ are the expectation and variance of *y*. The mean of *y* in the BMA is an average value obtained by weighting $\eta_{i}$ with $\omega_{i}$; its variance is the sum of the between-model variance $\left(\sum_{i\;=\;1}^{k}\omega_{i}{\left(\eta_{i}\;-\sum_{i\;=\;1}^{k}\omega_{i}\;\eta_{i}\right)}^{2}\;\right)$ and within-model variance $\left(\;\sum_{i\;=\;1}^{k}\omega_{i}\;\sigma_{i}^{2}\right)$. The between-model variance reflects the model selection error; the within-model variance refers to the error of the model itself.

BMA requires an accurate estimation of the weights and variances of each model in the model ensemble. The expectation–maximization algorithm is recommended for estimating the weights and variances of BMAs as it is simple to operate and satisfies that all BMA weights are nonnegative and sum to one (Raftery et al. 2005).

The EBMA was first proposed by Raftery et al. (2005) to integrate different forecasts and incorporate the prior uncertainty of the “best” model. Generally speaking, the EBMA generates predictions by creating a weighted average of the basic model or a predictive probability distribution function. The weight of the basic models is determined by two aspects: (i) the model with a more accurate prediction will obtain a greater weight, and (ii) the model with a unique and correct prediction will also obtain a greater weight. However, some predictions with too high an association may also have a larger weight and are penalized separately. The EBMA method creates an integrated prediction model based on the performance and uniqueness of multiple basic models used in previous predictions. This integrated model often outperforms the individual basic models. In this study, the EBMA method was used to integrate four basic models for the final estimation (Fig. 8C).

The models were implemented in the R package (v.4.2.2) using the “caret” package (https://cran.r-project.org/web/packages/caret/index.html) and “EBMAforecast” package (https://cran.r-project.org/web/packages/EBMAforecast/index.html) (Kuhn 2009). To tune the hyperparameters of the models, grid search and 5-fold inner cross-validation were used during the modeling process. The parameter ranges for grid search and final model hyperparameters are shown in Supplemental Table S5.

### Model performance evaluation

To assess the accuracy of the models, 80% of the samples were randomly selected as the training data set, and the remaining 20% were used as the testing data set. The modeling process was repeated 1,000 times to reduce errors, and the average of the results was used as the final estimation. The final data set used for the modeling step in this study included several predictors for both faba bean and pea crops. For faba bean, the predictors include PH, CC, NRCT, 8 Tis, and 25 VIs. The final data set for faba bean at a single growth stage consisted of 20 variables with 198 samples for RGB data, 23 variables with 198 samples for MS data, 9 variables with 198 samples for TIR data, 43 variables with 198 samples for RM data, 29 variables with 198 samples for RT data, 32 variables with 198 samples for MT data, and 52 variables with 198 samples for RMT data. Similarly, for pea, the final data set included the same predictors as faba bean. The final data set for pea at a single growth stage consisted of 20 variables with 132 samples for RGB data, 23 variables with 132 samples for MS data, 9 variables with 132 samples for TIR data, 43 variables with 132 samples for RM data, 29 variables with 132 samples for RT data, 32 variables with 132 samples for MT data, and 52 variables with 132 samples for RMT data. For the whole growth stage analysis, the final faba bean data set included 80 variables with 198 samples for RGB data, 92 variables with 198 samples for MS data, 36 variables with 198 samples for TIR data, 172 variables with 198 samples for RM data, 116 variables with 198 samples for RT data, 128 variables with 198 samples for MT data, and 208 variables with 198 samples for RMT data. The final pea data set for the whole growth stage analysis consisted of 80 variables with 132 samples for RGB data, 92 variables with 132 samples for MS data, 36 variables with 132 samples for TIR data, 172 variables with 132 samples for RM data, 116 variables with 132 samples for RT data, 128 variables with 132 samples for MT data, and 208 variables with 132 samples for RMT data. To evaluate the models, the faba bean data set was split into 158 training samples and 40 testing samples, while the pea data set was split into 105 training samples and 27 testing samples. This division allows for the training and testing of the models on independent samples to evaluate the model's performance and generalizability. The HI estimation accuracy of the models was evaluated using 4 indicators: the coefficient of determination (*R*^2^), root-mean-square error (RMSE), MAE, and NRMSE.


$$R^{2}\;=\;1-\;\overset{n}{\underset{i=1}{\sum }}\;(X_{i}-{\hat{X}}_{i}{)}^{2\;}/\;\overset{n}{\underset{i=1}{\sum }}\;(X_{i}-\bar{X}{)}^{2},$$ (8)

$$\mathrm{RMSE}\;=\;\sqrt{\;\overset{n}{\underset{i=1}{\sum }}\;{\left(X_{i}-{\hat{X}}_{i}\right)}^{2}\;/\;n},$$ (9)

$$\mathrm{MAE}\;=\;\overset{n}{\underset{i=1}{\sum }}\;|X_{i}-{\hat{X}}_{i}|\;/\;n,$$ (10)

$$\mathrm{NRMSE}\;=\;\mathrm{RMSE}\;/\;\bar{X},$$ (11)

where *n* is the total number of samples, $X_{i}$ and ${\hat{X}}_{i}$ are the measured and estimated HI of the samples, respectively, and $\bar{X}$ denotes the mean of the measured HI.

## Supplementary Material

kiad577_Supplementary_Data

## Supplemental data

The following materials are available in the online version of this article.

**Supplemental Table S1.** The details of the estimation results in faba bean.

**Supplemental Table S2.** The details of the estimation results in pea.

**Supplemental Table S3.** VI formula derived from UAV RGB images.

**Supplemental Table S4.** VI formula derived from UAV MS images.

**Supplemental Table S5.** Parameter ranges for grid search and final model hyperparameters.

**Supplemental Figure S1.** Scatter plots of measured and best estimated harvest index using EBMA model.
