# Supplementary material for: Faba bean and pea harvest index estimations using aerial-based multimodal data and machine learning algorithms
Source: Plant Physiol. 2023 Nov 3;194(3):1512–26. doi: 10.1093/plphys/kiad577 (PMC10904323; doi:10.1093/plphys/kiad577)
Supplement: kiad577_Supplementary_Data [file kiad577_supplementary_data.zip › Supplemental Figure S1.Scatter plots of measured and best estimated harvest index u.pdf]

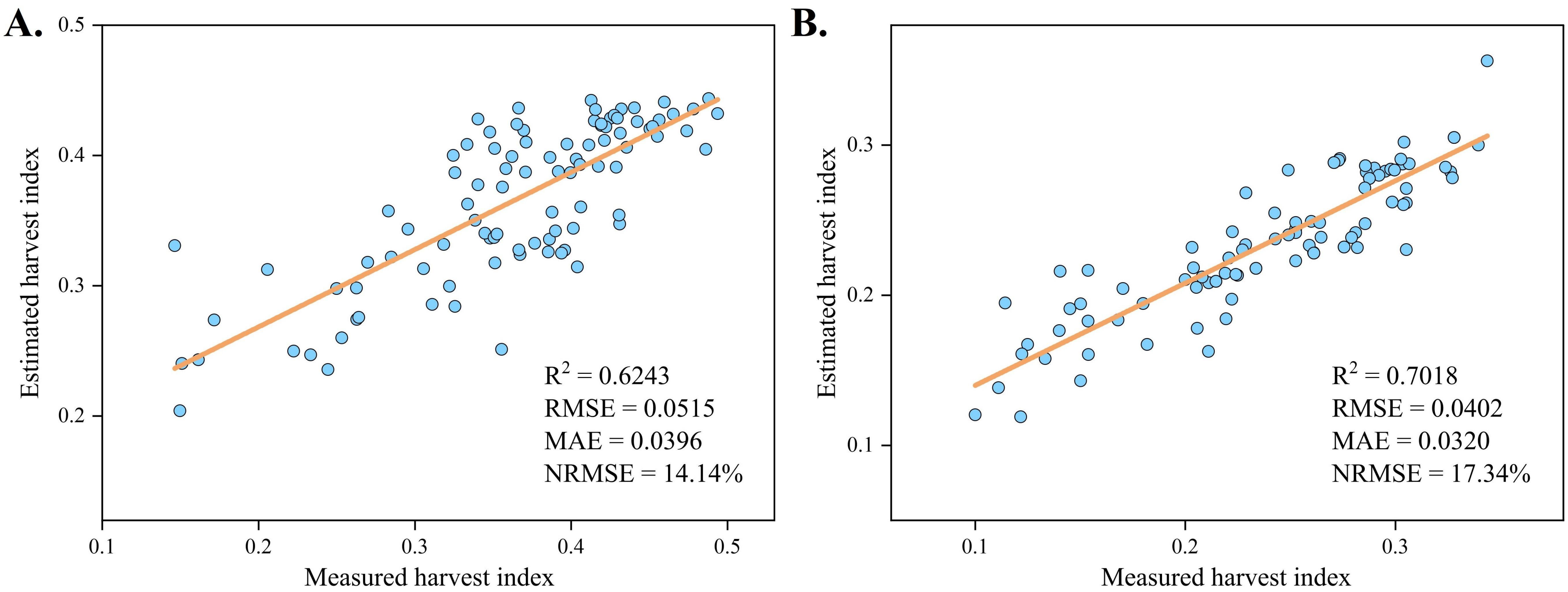

Figure S1. Scatter plots of measured and best estimated harvest index using ensemble Bayesian model averaging model. (A) The scatter plots of faba bean; (B) The scatter plots of pea.
